# The LncRNA MIR503HG/miR-224-5p/TUSC3 Signaling Cascade Suppresses Gastric Cancer Development *via* Modulating ATF6 Branch of Unfolded Protein Response 

**DOI:** 10.3389/fonc.2021.708501

**Published:** 2021-07-26

**Authors:** Han Lin, Jinge Wang, Tong Wang, Jiaming Wu, Peng Wang, Xiaoyan Huo, Jun Zhang, Huayang Pan, Yuying Fan

**Affiliations:** ^1^Department of General Surgery, Key Laboratory of Hepatosplenic Surgery, Ministry of Education, The First Affiliated Hospital of Harbin Medical University, Harbin, China; ^2^Department of Formulas of Chinese Medicine of Basic Medical College, Heilongjiang University of Chinese Medicine, Harbin, China; ^3^The Second Affiliated Hospital & College of Nursing, Harbin Medical University, Harbin, China

**Keywords:** unfolded protein response, gastric cancer, LncRNA MIR503HG, miR-224-5p, TUSC3

## Abstract

**Background:**

Unfolded protein response (UPR)-mediated tumor-promoting functions have been identified in multiple cancers, and this study focused on investigating the role and molecular mechanisms of UPR in modulating gastric cancer (GC) pathogenesis.

**Methods:**

The bioinformatics analysis was performed to examine the expression status of cancer associated genes in patients with stomach adenocarcinoma (STAD) and predict the targeting sites of miR-224-5p with LncRNA MIR503HG and TUSC3. Genes expressions were quantified by Real-Time qPCR, Western Blot and immunohistochemistry (IHC). Cell proliferation, viability, apoptosis and mobility were evaluated by MTT assay, trypan blue staining assay, flow cytometer and transwell assay, respectively. The binding sites were validated by dual-luciferase reporter gene system assay.

**Results:**

LncRNA MIR503HG and TUSC3 were downregulated, but miR-224-5p was upregulated in GC tissues and cells, in contrast with their normal counterparts. Further gain- and loss-of-function experiments validated that the malignant phenotypes in GC cells, including cell proliferation, invasion, epithelial-mesenchymal transition (EMT) and tumorigenesis, were negatively regulated by LncRNA MIR503HG. Mechanistically, LncRNA MIR503HG upregulated TUSC3 in GC cells through sponging miR-224-5p, resulting in the repression of GC progression. Finally, we validated that knock-down of ATF6, but not other two branches of UPR (PERK1 and IRE1), partially rescued cell proliferation and EMT in the GC cells with LncRNA MIR503HG overexpression.

**Conclusions:**

Targeting the LncRNA MIR503HG/miR-224-5p/TUSC3 signaling cascade suppressed ATF6-mediated UPR, resulting in the blockage of GC development.

## Background

Identification of cancer associated biomarkers has been proved as a reasonable strategy to provide novel targets for gastric cancer (GC) diagnosis, prognosis and therapy (1, 2), and the long non-coding RNAs (LncRNAs) are closely associated with GC pathogenesis. Those LncRNAs, including LncRNA MEG3 (3), LncRNA PVT1 (4), LncRNA AK023391 (5), and LncRNA HOXA11-AS (6), play important roles in GC. Interestingly, their biological functions in regulating GC progression vary according to differential types of LncRNAs, and they act as both tumor suppressors (3, 7, 8) and oncogenes (4–6) in GC. Among all the LncRNAs, LncRNA MIR503 host gene (MIR503HG) is located on chromosome Xq26.3, and dysregulated LncRNA MIR503HG contributes to the aggressiveness of multiple cancers (9–11). Specifically, researcher notice that LncRNA MIR503HG functions as a tumor suppressor to restrain the development breast cancer (10), bladder cancer (11), and colorectal cancer (9), but it is still unclear whether LncRNA MIR503HG involves in regulating GC progression, thus, we selected LncRNA MIR503HG for further investigations in the present study.

According to the principles of competing endogenous RNA (ceRNA) network mechanisms, LncRNAs exert their biological functions through serving as RNA spongers for microRNAs (miRNAs) (12, 13). Our preliminary bioinformatics analysis (data not shown) screened out miR-224-5p as the downstream target for LncRNA MIR503HG, thus, miR-224-5p was selected for further analysis. Interestingly, according to the information provided by the previous work, the role of miR-224-5p in regulating GC progression is controversial, and miR-224-5p is validated as tumor suppressor by Li et al. (14), but is identified as oncogene by Fang et al. (15) in GC. Therefore, it is necessary to assure the regulating effects of miR-224-5p on GC progression. In addition, as previously reported (16), tumor suppressor candidate 3 (TUSC3) is identified as the downstream target of miR-224-5p. Notably, TUSC3 has dual-functions in regulating cancer progression according to different cancer types (17), on the one hand, Kong et al. evidence that upregulation of TUSC3 inhibits proliferation and induces apoptosis in retinoblastoma (18), while Gu et al. find that TUSC3 promotes cancer development in colorectal cancer (19). Although Yuasa et al. try to investigate the association of TUSC3 methylation with GC (20), the detailed mechanistic information are still not fully delineated.

Unfolded protein response (UPR) is activated in response to endoplasmic reticulum (ER) stress, leading to protein translation repression, improvement of ER protein folding and clearance capacity (21–23), which allows tumor cells to restore ER proteostasis and facilitates cancer cells to adapt to the restrictive microenvironments with nutrient and oxygen deprivation, and promotes cancer development (24–26). The activation of UPR can be mediated by three branches of signaling cascades, including PERK, IRE1 and ATF6, respectively (25, 27, 28). Mechanistically, the unfolded proteins in the ER of the cells attract the molecular chaperon BIP, which led to the release of the PERK, IRE1 and ATF6 proteins on the ER membrane to initiate UPR process (25, 27, 28). Of note, the ATF6-dependent UPR exerts cyto-protective effects to accelerate cancer progression and drug resistance. Interestingly, TUSC3 loss alters ER stress to facilitate prostate cancer progression (29), and recent data evidence that TUSC3-deficiency enhances ATF6-mediated UPR and metastatic potential to aggravate cancer development in non-small cell lung cancer (16), but the detailed mechanisms have not been fully delineated.

The present study, for the first time, reported the involvement of the LncRNA MIR503HG/miR-224-5p/TUSC3 signaling cascade in regulating GC pathogenesis, and the potential underlying mechanisms had also been uncovered, which provided evidences to support that this axis could be used as potential biomarkers for GC diagnosis and treatment.

## Methods

### Clinical Specimens

The GC patients (N = 42, 14 male and 28 female, aged from 30 to 67 years old) were recruited in the First Affiliated Hospital of Harbin Medical University from 2017 to 2019, and the cancerous and adjacent non-cancerous tissues were collected from those participants and were immediately frozen at -80°C refrigerator for further analysis. We assured that all the GC patients did not accept any other treatment strategies, such as chemo- or radio-therapy before surgical resection, and two experienced pathologists were invited to judge the TNM stage and lymphatic migration status of the patients. All the patients had signed the informed consent forms, and the clinical experiments were approved by the Ethics Committee of the First Affiliated Hospital of Harbin Medical University.

### Cell Culture and Vectors Transfection

The gastric cancer cell lines (SGC7901 and BGC-823) and human normal gastric epithelial cell line GES-1 were obtained from Cancer Research Institute of Beijing (China), and all the cells were maintained in the Dulbecco’s Modified Eagle Medium (DMEM, Gibco, USA) with 10% fetal bovine serum (FBS, Gibco, USA). The cells were incubated in an incubator with 5% CO_2_ atmosphere and temperature at 37°C. The overexpression and downregulation vectors involved in this study were designed by Sangon Biotech (Shanghai, China) according to the sequences provided by the previous publications (9, 15, 16, 19). All the vectors were delivered into the GC cells by using the commercial Lipofectamine 3000 transfection kit (Invitrogen, USA) according to manufacturer’s protocol.

### Real-Time qPCR

The expression levels of LncRNA MIR503HG, miR-224-5p, TUSC3 mRNA, PERK mRNA, IRE1 mRNA and ATF6 mRNA were quantified by using the Real-Time qPCR analysis. Briefly, the total RNA was extracted from the GC tissues and cells by Trizol reagent (Takara, USA) following manufacturer’s instruction. The RT MasterMix (Abcam, UK) was used to reversely transcribed into cDNA, which were examined by agarose electrophoresis. Next, the expression levels of the above genes were examined by the SYBR green I (Roche Diagnostics, Switzerland), and miR-224-5p was normalized by U6, and other genes were normalized by GAPDH. The primer sequences for Real-Time qPCR could be found in the previous publications (9, 15, 16, 19).

### Western Blot Analysis

The total proteins were extracted by using the RIPA lysis buffer according to manufacturer’s protocols, and the protein bands were separated according to molecular weight (MW) by using the following SDS-PAGE. Then, the target proteins were transferred onto PVDF membranes (Millipore, USA), the membranes were blocked by 5% slim milk and were incubated with the primary antibodies against TUSC3 (1:1500, Abcam, UK), N-cadherin (1:2000, Abcam, UK), Vimentin (1:2000, Abcam, UK) and GAPDH (1:3000, Takara, USA) at 4°C overnight. After that, the membranes were probed with the anti-rabbit secondary antibody (1:3000, Takara, USA) for 1.5 h at room temperature. Finally, the ECL system (ThermoFisher Scientific, USA) was employed to visualize the protein bands, which were quantified by the Image J software. The relative expression levels of the proteins were normalized by GAPDH.

### Examination of Cell Proliferation and Viability

The GC cells with differential vectors transfection were cultured in the 96-well plates for 0 h, 24 h and 48 h, and MTT assay and trypan blue staining assay were conducted to examine cell proliferation and viability, respectively. For MTT assay, the GC cells were incubated with MTT solution for 2 h at 37°C, which were subsequently vortexed and a microplate reader (ThermoFisher Scientific, USA) was used to examine optical density (OD) values at the wavelength of 450 nm, which could be used to evaluate cell proliferation abilities. For trypan blue staining assay, the GC cells were stained with trypan blue staining dye for 30 min at t 37°C, and a light microscope was used to observe and count the number of dead blue cells, and cell viability was calculated by using the following formula: viability (%) = (total cells – dead blue cells)/total cells × 100%.

### Transwell Assay

The GC cells were cultured in the upper chambers of the transwell plates (Corning, USA) with serum-free DMEM medium, and the lower chambers were full of DMEM medium containing 10% FBS as chemoattractant. At 24 h post-culture under standard culture conditions, the cells on the upper surface of the Matrigel were washed and removed by PBS buffer, and the lower surface was stained with 0.1% crystal violet to visualize the cells. Finally, a light microscope was used to photograph, observe and count the number of the cells, which could reflect the invasion abilities of the GC cells.

### Dual-Luciferase Reporter Gene System Assay

The online starBase software (http://starbase.sysu.edu.cn/) was used to predict the targeting sites of miR-224-5p with LncRNA MIR503HG and 3’ UTR of TUSC3 mRNA, which were subsequently mutated and cloned into the luciferase plasmids by the commercial third-party company (Sangon Biotech, Shanghai, China). After that, the mimic for miR-224-5p, and the luciferase vectors were co-delivered into the GC cells. At 48 h post-transfection, the luciferase reporter assay kit (Promega, USA) and a luminescence plate reader (Molecular Devices, USA) were employed to examine the relative luciferase activities in the above cells. The activities of the firefly luciferase was normalized by the Renilla luciferase.

### RNA Pull-Down Assay

The biotin-labelled LncRNA MIR503HG and 3’UTR of TUSC3 mRNA probes were designed and constructed by Sangon Biotech (Shanghai, China), and the interactions of miR-224-5p with LncRNA MIR503HG and TUSC3 were measured by performing the following RNA pull-down assay. The GC cells were lysed and sonicated, which were subsequently subjected to centrifugation, and the supernatants were collected and a small fraction of them was prepared as input. Then, the rest of the supernatants were incubated with the LncRNA MIR503HG and TUSC3 probes combined M-280-streptavidin Dynabeads (Invitrogen, USA) at 30°C overnight, and the probes-miR-224-5p complex was formed, and the formaldehyde crosslinking was released by incubating the mixtures with lysis buffer and Proteinase K. The enrichment of miR-224-5p was evaluated by using the Real-Time qPCR analysis.

### Flow Cytometer Analysis for Cell Apoptosis

The GC cells were double-stained with Annexin V-FITC and PI dyes for 45 min at 37°C without light exposure by using the commercial Apoptosis Detection Kit purchased from YEASEN (Shanghai, China). After that, the flow cytometer (Becton-Dickinson, USA) was used to examine cell apoptosis ratio. The cells stained with either of Annexin V-FITC or PI were taken into consideration as apoptotic cells.

### *In Vivo* Animal Experiments

The male Balb/c nude mice (N = 10) were obtained from the Research Animal Center of Harbin Medical University and were fed in the specific-pathogen-free (SPF) conditions with freely accessible to food and water. The SGC7901 cells with or without LncRNA MIR503HG were injected into the dorsal flank of the mice, and tumor formation status were monitored every 5 days from day 0 to day 25 post-injection. At day 25, the mice were anesthetized by pentobarbital sodium (50 mg/kg) and were photographed. Then, the mice were sacrificed and the tumor tissues were obtained by surgical resection for further analysis. All the animal experiments had been approved by the Ethics Committee in the First Affiliated Hospital of Harbin Medical University.

### Examination of Ki67 Expressions and Localization by Immunohistochemistry (IHC)

The mice tumor tissues were prepared as sections with about 5 μm thickness, which were fixed by 10% (v/v) formaldehyde and were embedded by paraffin. According to the experimental protocols provided by the previous studies (30, 31), the IHC assay was conducted to examine the expression levels and localization of Ki67 protein in the mice tumor tissues. The primary antibody against Ki67 protein was purchased from Takara (USA) at the working concentration of 1:3000, and the light microscope was used to observe and photograph the yellow Ki67-positive cells in the mice tissues.

### Collection, Analysis and Visualization of the Data

All the data were collected and presented as Means ± Standard Deviation (SD), and were analyzed by SPSS 18.0 software. Comparisons between two groups were conducted by the Student’s t-test, and the one-way ANOVA analysis was used to compare the means from multiple groups. Individual experiment had three repetitions, and *P* < 0.05 was marked by “*”.

## Results

### The Correlations of LncRNA MIR503HG, miR-224-5p and TUSC3 With GC Progression and Prognosis

**Figure 1:**
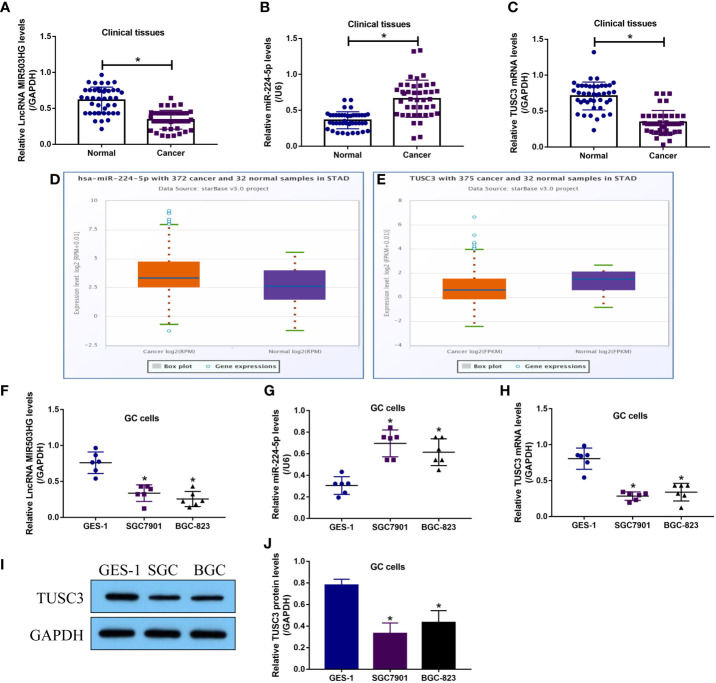
LncRNA MIR503HG, miR-224-5p and TUSC3 were aberrantly expressed in GC tissues and cells. The expression levels of **(A)** LncRNA MIR503HG, **(B)** miR-224-5p and **(C)** TUSC3 mRNA in GC tissues were examined by using the Real-Time qPCR analysis. Pan-cancer analysis was performed to analyze the expressions of **(D)** miR-224-5p and **(E)** TUSC3 in the normal and cancer tissues of STAD patients. The expression status of **(F)** LncRNA MIR503HG, **(G)** miR-224-5p, and TUSC3 **(H)** mRNA and **(I, J)** protein were analyzed by Real-Time qPCR and Western Blot, respectively. Each experiment contained 3 repetitions, and *P* < 0.05 were marked by “*”.

Initially, we investigated the expression status of LncRNA MIR503HG, miR-224-5p and TUSC3 in GC tissues and cells, and the Real-Time qPCR results showed that LncRNA MIR503HG (**Figure 1A**) and TUSC3 mRNA (**Figure 1C**) were downregulated, while miR-224-5p (**Figure 1B**) was significantly upregulated in the cancerous tissues, in contrast with the corresponding adjacent normal tissues collected from GC patients (N = 42). The above results were supported by the following Pan-cancer analysis (http://starbase.sysu.edu.cn/), which indicated that miR-224-5p tended to be enriched, while TUSC3 was downregulated in the cancer tissues (N = 375), compared to the normal samples (N = 32), collected from patients with stomach adenocarcinoma (STAD) (**Figures 1D, E**). Next, the correlations of the above cancer associated genes with clinical parameters were analyzed, the results showed that LncRNA MIR503HG (**Figures S1A–E**) and TUSC3 (**Figures S3A–E**) were significantly downregulated, but miR-224-5p (**Figures S2A–E**) was high-expressed in patients with tumor size (> 3), TNM stage (III/IV) and lymphatic metastasis (yes), but had nothing to do with patients’ age and gender. In addition, the GC cells (SGC7901 and BGC-823) and normal GES-1 cells were examined by Real-Time qPCR (**Figures 1F–H**) and Western Blot analysis (**Figures 1I, J**), and the results verified that the expression levels of LncRNA MIR503HG and TUSC3 were lower, while miR-224-5p was higher in the GC cells, compared to the GES-1 cells (**Figures 1F–J**).

### LncRNA MIR503HG Acted as a Tumor Suppressor to Hinder the Development of GC *In Vitro* and *In Vivo*

**Figure 2:**
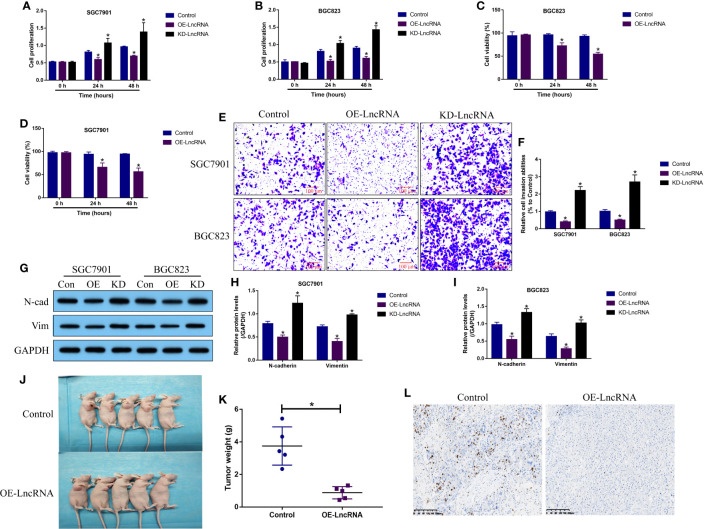
LncRNA MIR503HG functioned as a tumor suppressor to reverse the malignant phenotypes in GC cells. **(A, B)** Cell proliferation abilities were analyzed by MTT assay, and further **(C, D)** trypan blue staining assay was performed to evaluate cell viability. **(E, F)** Cell invasion abilities were determined by using the transwell assay. **(G–I)** The Western Blot analysis was conducted to examine the expression levels of N-cadherin and Vimentin in GC cells, which were normalized by the GAPDH. “OE” represented “LncRNA MIR503HG overexpression”, and “KD” suggested “LncRNA MIR503HG ablation”. **(J, K)** The xenograft tumor bearing mice models were established by using the SGC7901 cells, and tumor weight were obtained. **(L)** Immunohistochemistry (IHC) assay supported that LncRNA MIR503HG downregulated Ki67 in mice tumor tissues. Each experiment contained 3 repetitions, and *P* < 0.05 were marked by “*”.

LncRNA MIR503HG acts as a tumor suppressor to hamper the development of multiple cancers (9–11), and we validated that LncRNA MIR503HG also exerted its tumor-inhibiting effects in GC. Functionally, the GC cells (SGC7901 and BGC-823) with LncRNA MIR503HG overexpression and downregulation were established (**Figure S4A**), and the MTT assay results in **Figures 2A, B** showed that LncRNA MIR503HG negatively regulated cell proliferation in GC cells in a time-dependent manner. Specifically, LncRNA MIR503HG overexpression suppressed, while LncRNA MIR503HG downregulation promoted cell proliferation in both SGC7901 and BGC823 cells. Next, the trypan blue staining assay was performed, and we confirmed that overexpression of LncRNA MIR503HG also suppressed cell viability in GC cells (**Figures 2C, D**). Also, we performed transwell assay to determine cell invasion abilities, and the results showed that GC cell invasion was inhibited by overexpressing LncRNA MIR503HG, and conversely, knock-down of LncRNA MIR503HG facilitated cell mobility (**Figures 2E, F**). Then, by performing further Western Blot analysis, we found that LncRNA MIR503HG overexpression decreased the expression levels of N-cadherin and Vimentin to hamper EMT process in GC cells, while LncRNA MIR503HG ablation had opposite effects on EMT (**Figures 2G–I**). Finally, the SGC7901 cells with or without LncRNA MIR503HG overexpression were utilized to establish xenograft tumor-bearing mice models. As expected, LncRNA MIR503HG inhibited GC tumor growth *in vivo* (**Figures 2J, K**). Consistently, the immunohistochemistry (IHC) assay results supported that LncRNA MIR503HG overexpression decreased Ki67 protein levels in mice tumor tissues (**Figure 2L**).

### The Regulating Mechanisms of LncRNA MIR503HG, miR-224-5p and TUSC3 in GC Cells

**Figure 3:**
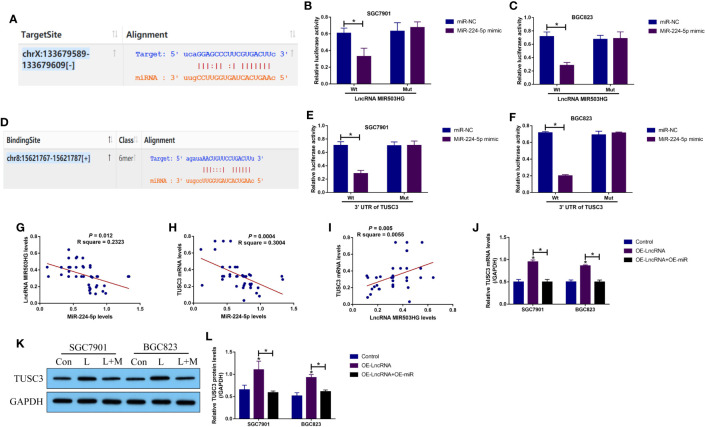
The regulatory mechanisms of LncRNA MIR503HG, miR-224-5p and TUSC3 in GC cells. The targeting sites of miR-224-5p with **(A–C)** LncRNA MIR503HG and **(D–F)** 3’ UTR of TUSC3 mRNA were predicted by the online starBase software (http://starbase.sysu.edu.cn/) and were validated by the following dual-luciferase reporter gene system assay. The activities of the firefly luciferase was normalized by the Renilla luciferase. **(G–I)** The correlations of LncRNA MIR503HG, miR-224-5p and TUSC3 in GC tissues were analyzed. **(J)** Real-Time qPCR and **(K, L)** Western Blot were performed to determine TUSC3 expressions in GC cells. “L” represented “LncRNA MIR503HG overexpression”, and “L+M” indicated “OE- LncRNA MIR503HG+OE-miRNA”. Each experiment contained 3 repetitions, and *P* < 0.05 were marked by “*”.

Next, by performing the bioinformatics analysis, we noticed that there existed binding sites in miR-224-5p with LncRNA MIR503HG (**Figure 3A**) and 3’ untranslated regions (3’UTRs) of TUSC3 mRNA (**Figure 3D**). According to the principles of competing endogenous RNA (ceRNA) mechanisms, we conjectured that there might exist regulating mechanisms among LncRNA MIR503HG, miR-224-5p and TUSC3. By performing the following dual-luciferase reporter gene system assay, we validated that miR-224-5p targeted both LncRNA MIR503HG (**Figures 3B, C**) and 3’ UTR of TUSC3 mRNA (**Figures 3E, F**), which were supported by the RNA pull-down assay results that miR-224-5p could be enriched by both biotin-labelled LncRNA MIR503HG (**Figures S6A, B**) and TUSC3 (**Figures S6C, D**) in GC cells. Then, we conducted Pearson correlation analysis to analyze the correlations of LncRNA MIR503HG, miR-224-5p and TUSC3 mRNA levels in GC tissues, and the results showed that miR-224-5p negatively correlated with LncRNA MIR503HG (**Figure 3G**) and TUSC3 mRNA (**Figure 3H**), but LncRNA MIR503HG and TUSC3 mRNA showed positive relevance in GC tissues (**Figure 3I**). Subsequently, the LncRNA MIR503HG overexpression vectors (**Figure S4A**) and miR-224-5p mimic (**Figure S4B**) were delivered into the GC cells, which were divided into three groups as follows: Control, OE-LncRNA MIR503HG group, and OE-LncRNA MIR503HG + OE-miR-224-5p group. As expected, the results in **Figures 3J–L** showed that LncRNA MIR503HG upregulated TUSC3 in GC cells at both transcriptional and translational levels, which were reversed by overexpressing miR-224-5p. In addition, the regulatory effects among LncRNA MIR503HG, miR-224-5p and TUSC3 were discussed, as shown in **Figures S7A, B**, overexpression and downregulation of LncRNA MIR503HG did not affect miR-224-5p expressions (**Figure S7A**), and conversely, manipulation of miR-224-5p also had little effects on LncRNA MIR503HG expressions (**Figure S7B**), suggesting that LncRNA MIR503HG merely acted as a RNA sponger for miR-224-5p, and miR-224-5p itself did not influence LncRNA MIR503HG expressions. Moreover, the mRNA levels of TUSC3 were positively regulated by LncRNA MIR503HG (**Figure S7C**), and were negatively regulated by miR-224-5p (**Figure S7D**).

### Upregulation of LncRNA MIR503HG Induced GC Cell Death and Inhibited EMT Through Modulating the miR-224-5p/TUSC3 Axis

**Figure 4:**
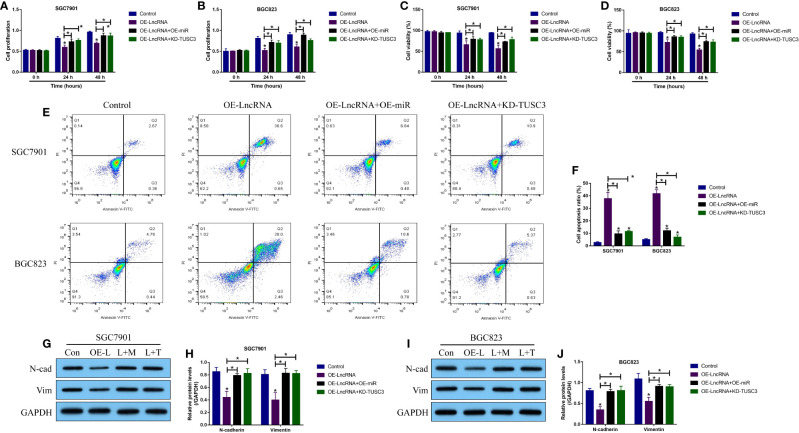
LncRNA MIR503HG exerted its tumor-inhibiting effects by regulating the miR-224-5p/TUSC3 axis. **(A, B)** Cell proliferation and **(C, D)** viability were examined by using the MTT assay and trypan blue staining assay, respectively. **(E, F)** The GC cells were stained with Annexin V-FITC and PI, and the FCM assay was used to examine cell apoptosis ratio. **(G–J)** The expression levels of N-cadherin and Vimentin were detected by Western Blot analysis. “OE-L” indicated “LncRNA MIR503HG overexpression”, “L+M” suggested “OE-LncRNA MIR503HG + OE-miRNA”, and “L + T” represented “OE-LncRNA MIR503HG + KD-TUSC3”. Each experiment contained 3 repetitions, and *P* < 0.05 were marked by “*”.

Given that the LncRNA MIR503HG-miR-224-5p-TUSC3 ceRNA network had been identified in GC cells, we next purposed that LncRNA MIR503HG regulated the malignant phenotypes of GC cells by regulating the miR-224-5p/TUSC3 axis. Hence, the LncRNA MIR503HG overexpression vectors (OE-LncRNA MIR503HG) (**Figure S4A**), miR-224-5p mimic (OE-miR-224-5p) (**Figure S4B**) and TUSC3 knock-down vectors (KD-TUSC3) (**Figure S4C**) were transfected into the GC cells, which were divided into four groups, including Control, OE-LncRNA MIR503HG group, OE-LncRNA MIR503HG + OE-miR-224-5p group, and OE-miR-224-5p + KD-TUSC3 group. As shown in **Figures 4A–D**, the MTT assay and trypan blue staining assay results showed that both miR-224-5p overexpression and TUSC3 silence rescued cell proliferation and viability in GC cells with LncRNA MIR503HG overexpression. In addition, the flow cytometer was used to detect cell apoptosis, and as expected, overexpression of LncRNA MIR503HG increased apoptosis ratio in GC cells, which were partially reversed by overexpressing miR-224-5p and downregulating TUSC3 (**Figures 4E, F**). Also, data in **Figures 4G–J** showed that the inhibiting effects of LncRNA MIR503HG on EMT were abrogated by both miR-224-5p overexpression and TUSC3 downregulation.

### LncRNA MIR503HG Overexpression Suppressed Cell Viability and EMT in GC Cells in a ATF6-Mediated UPR Dependent Manner

**Figure 5:**
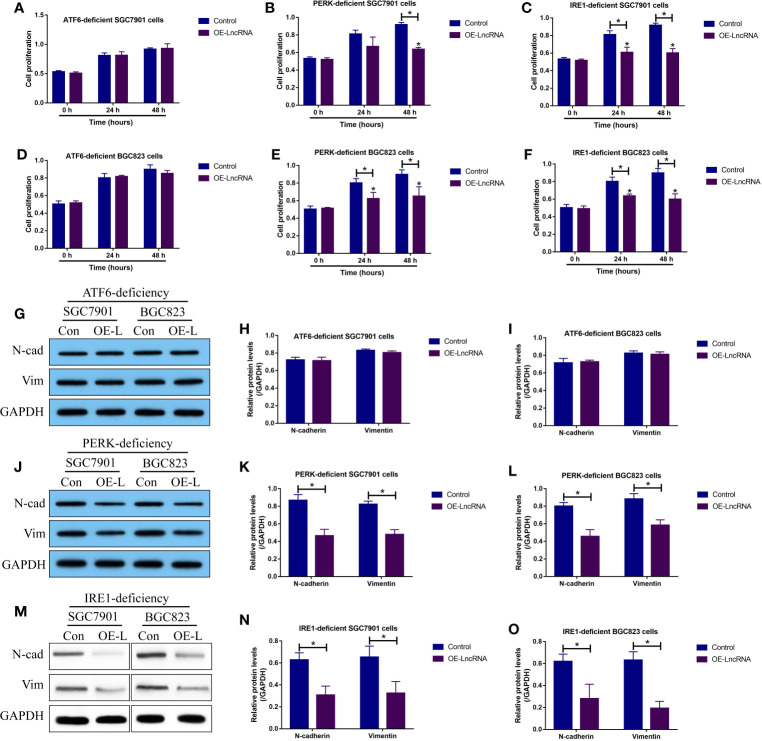
LncRNA MIR503HG inhibited cell proliferation and EMT in GC cells by modulating ATF6-mediated UPR. The PERK-, IRE1- and ATF6-deficient SGC7901 and BGC823 cells were used, and **(A–F)** cell proliferation abilities were evaluated by using the MTT assay. **(G–O)** The expression status of N-cadherin and Vimentin were examined by Western Blot analysis. “OE-L” represented “LncRNA MIR503HG overexpression”. Each experiment contained 3 repetitions, and *P* < 0.05 were marked by “*”.

Although the LncRNA MIR503HG/miR-224-5p/TUSC3 axis was proved to be crucial for regulating GC progression, the downstream mechanisms had not been investigated. Previous data suggested that ER stress mediated UPR contributes to cancer metastasis, and TUSC3-deficiency enhanced UPR to promote metastatic potential of non-small cell lung cancer (NSCLC) (16), which encouraged us to investigate whether LncRNA MIR503HG inhibited GC metastasis in a UPR-dependent manner. To explore this issue, according to the protocols provided by the previous work (16), the three branches of UPR, including PERK, IRE1 and ATF6, were silenced in GC cells, respectively (**Figures S5A–C**). Next, the GC cells with PERK-, IRE1- and ATF6-deficiency were co-transfected with LncRNA MIR503HG overexpression vectors (**Figure S4A**). The MTT assay results showed that upregulation of LncRNA MIR503HG significantly inhibited cell proliferation in PERK- and IRE1- deficient GC cells, but had little effects on the cell proliferation abilities of GC cells with ATF6-deficiency (**Figures 5A–F**). In addition, the Western Blot analysis was conducted to examine EMT associated biomarkers, and the results in **Figures 5G–O** supported that LncRNA MIR503HG inhibited N-cadherin and Vimentin expressions in GC cells without PERK and IRE1 expressions, but did not influence EMT in ATF6-deficient GC cells.

## Discussion

Up until now, various cancer-associated genes, including tumor suppressors and oncogenes, have been identified as diagnostic and prognostic biomarkers for gastric cancer (GC) (1, 2), which shed light on the discovery of effective treatment strategies for this disease. Among all the signal pathways that regulate cancer development, the LncRNAs-miRNAs-mRNAs competing endogenous networks are closely associated with the pathogenesis of GC (3–6), and this study, for the first time, identified a novel LncRNA MIR503HG/miR-224-5p/TUSC3 signaling cascade that regulated unfolded protein response (UPR) to hamper the development of GC. Specifically, we noticed that LncRNA MIR503HG was aberrantly downregulated in GC cells and tissues, and further experiments validated that LncRNA MIR503HG served as a tumor suppressor to reverse the malignant phenotypes and suppress aggressiveness of GC cells *in vitro* and *in vivo*, which are supported by the data from other teams in breast cancer (10), bladder cancer (11), and colorectal cancer (9).

Then, the principles of ceRNA network mechanisms (12, 13) convince us to investigate the downstream targets of LncRNA MIR503HG, and we successfully screened out that LncRNA MIR503HG sponged miR-224-5p to upregulate TUSC3 in GC cells, and both miR-224-5p overexpression and TUSC3 ablation abrogated the tumor-inhibiting effects of LncRNA MIR503HG on GC, implying that LncRNA MIR503HG regulated the miR-224-5p/TUSC3 axis to hamper GC progression. Of note, miR-224-5p has been identified as both tumor suppressor (14) and oncogene (15) in GC, which is controversial and brings puzzlements for researchers in this field. Our results supported the work conducted by Fang et al. (15) and verified that miR-224-5p played an tumor-promoting role in GC. In addition, the role of TUSC3 in regulating cancer progression varies according to cancer types (18, 19), but its role in regulating GC progression has not been reported. Our data solved this academic issue, and provided evidences to support that TUSC3 acted as a tumor suppressor to inhibit GC progression.

Endoplasmic reticulum (ER) stress mediated UPR allows tumor cells to restore ER proteostasis and facilitate cancer cells to adapt to the restrictive microenvironments with nutrient and oxygen deprivation (21–23), resulting in the aggressiveness of multiple cancers (24–26), including GC (32–34). To our knowledge, there exist three branches of signaling pathways that trigger UPR, including PERK, IRE1 and ATF6-branches (21–23), respectively. Interestingly, according to the information from the previous publications, TUSC3 exerts its regulating effects on UPR, and TUSC3-deficiency specifically activates ATF6-branch mediated UPR to promote cancer development and metastasis in prostate cancer (29) and non-small cell lung cancer (16). Given the fact that LncRNA MIR503HG positively regulates TUSC3 in GC cells, we validated that LncRNA MIR503HG inhibited cell proliferation and epithelial-mesenchymal transition (EMT) in PERK- and IRE1-deficient GC cells, but had little effects on the GC cells with ATF6-deficiency, suggesting that LncRNA MIR503HG inhibited GC progression in a ATF6-mediated UPR dependent manner, and our results were partially supported by the previous work (32–34). However, it was still unclear whether the LncRNA MIR503HG/miR-224-5p/TUSC3 signaling cascade directly regulated ATF6 expressions and activation, and the detailed mechanisms by which LncRNA MIR503HG regulated UPR were still needed to be uncovered in our future work.

## Conclusions

**Figure 6:**
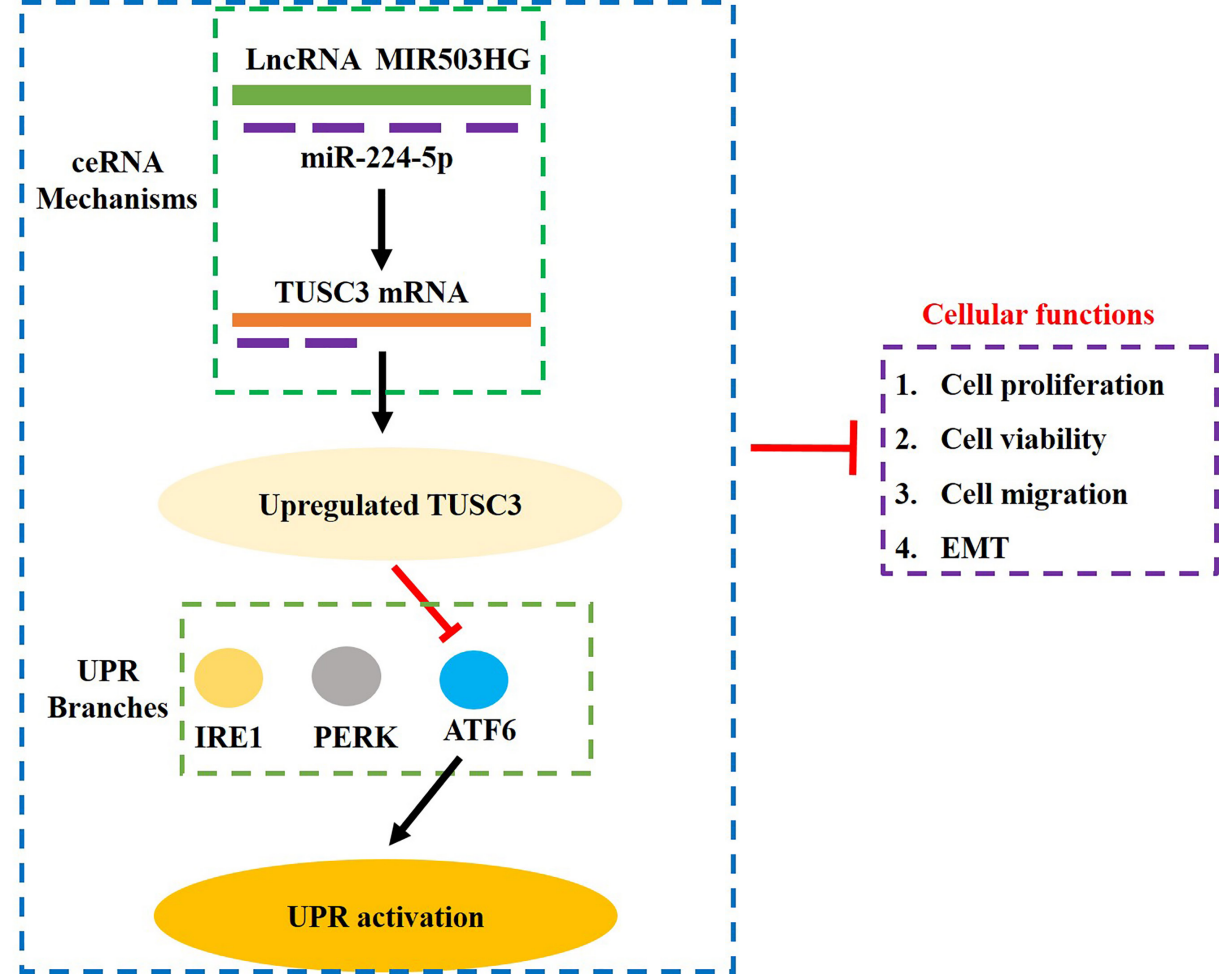
The graphical abstract of this study. Briefly, overexpression of LncRNA MIR503HG sponged miR-224-5p to upregulate TUSC3, which further specifically inactivated the ATF6 branch of UPR, resulting in the inhibition of proliferation, viability, Invasion and EMT in GC cells.

Taken together, analysis of the data indicated that LncRNA MIR503HG sponged miR-224-5p to upregulate TUSC3, leading to the suppression of ATF6 branch of UPR and GC development, which further suppressed the aggressiveness of GC (**Figure 6**). Our study, for the first time, validated that targeting the LncRNA MIR503HG/miR-224-5p/TUSC3 signaling cascade mediated UPR inactivation could be used as potential treatment strategy for GC.

## Data Availability Statement

The original contributions presented in the study are included in the article/**Supplementary Material**. Further inquiries can be directed to the corresponding authors.

## Ethics Statement

The studies involving human participants were reviewed and approved by Ethics Committee of the First Affiliated Hospital of Harbin Medical University. The patients/participants provided their written informed consent to participate in this study. The animal study was reviewed and approved by the Ethics Committee in the First Affiliated Hospital of Harbin Medical University.

## Author Contributions

HL, JWa, and TW designed this study, they were responsible for conducting the investigations and manuscript drafting. JWu, PW, XH, and JZ provided technical support, and they also collected, analyzed and visualized the involved data. HP and YF were corresponding authors, they provided conception, guidance, funding, manuscript validation and submission for this work. All authors contributed to the article and approved the submitted version.

## Funding

The University Nursing Program for Young Scholars with Creative Talents in Heilongjiang Province (UNPYSCT-2017064), acquired by HL. The Heilongjiang Postdoctoral Foundation (LBH-Z20196), acquired by HL. The Scientific Foundation of the First Affiliated Hospital of Harbin Medical University (2014B15), acquired by HP. The University Nursing Program for Young Scholars with Creative Talents in Heilongjiang Province (UNPYSCT-2018070), acquired by HP. The Heilongjiang Postdoctoral Scientific Research Developmental Fund (LBH-Q18089), acquired by HP. The Scientific Research Project of Health and Family Planning Commission of Heilongjiang Province (2014–303), acquired by HP. National College Students Innovation and Entrepreneurship Training Program (202010226016), acquired by HP. The role of all the funders is to provide financial supports and resources for this work.

## Conflict of Interest

The authors declare that the research was conducted in the absence of any commercial or financial relationships that could be construed as a potential conflict of interest.

## Publisher’s Note

All claims expressed in this article are solely those of the authors and do not necessarily represent those of their affiliated organizations, or those of the publisher, the editors and the reviewers. Any product that may be evaluated in this article, or claim that may be made by its manufacturer, is not guaranteed or endorsed by the publisher.

## Supplementary Material

Supplementary Figure 1 Analysis of the correlations of LncRNA MIR503HG and the clinical characteristics in GC patients by Real-Time qPCR. Each experiment contained 3 repetitions, and *P* < 0.05 were marked by "*".

Supplementary Figure 2 Analysis of the correlations of miR-224-5p and the clinical characteristics in GC patients by Real-Time qPCR. Each experiment contained 3 repetitions, and *P* < 0.05 were marked by "*".

Supplementary Figure 3 Analysis of the correlations of TUSC3 mRNA and the clinical characteristics in GC patients by Real-Time qPCR. Each experiment contained 3 repetitions, and *P* < 0.05 were marked by "*".

Supplementary Figure 4 LncRNA MIR503HG, miR-224-5p and TUSC3 were downregulated and overexpressed in GC cells, examined by Real-Time qPCR analysis. Each experiment contained 3 repetitions, and *P* < 0.05 were marked by "*".

Supplementary Figure 5 Real-Time qPCR was used to determine that ATF6, PERK and IRE1 were successfully deleted in the GC cells. Each experiment contained 3 repetitions, and *P* < 0.05 were marked by "*".

Supplementary Figure 6 RNA pull-down assay was performed to verify the targeting sites among LncRNA MIR503HG, miR-224-5p and 3’UTR of TUSC3 in GC cells. Each experiment contained 3 repetitions, and *P* < 0.05 were marked by "*".

Supplementary Figure 7 The regulating effects among LncRNA MIR503HG, miR-224-5p and TUSC3 were measured by Real-Time qPCR analysis. The regulating effects of LncRNA MIR503HG on **(A)** miR-224-5p and **(C)** TUSC3 mRNA expressions. MiR-224-5p was overexpressed and downregulated, and its effects on **(B)** LncRNA MIR503HG and **(D)** TUSC3 mRNA were evaluated. Each experiment contained 3 repetitions, and *P* < 0.05 were marked by "*".

## Abbreviations

UPR, unfolded protein response; GC, gastric cancer; STAD, stomach adenocarcinoma; IHC, immunohistochemistry; EMT, epithelial-mesenchymal transition; LncRNAs, long non-coding RNAs; ceRNA, competing endogenous RNA; miRNAs, microRNAs; TUSC3, tumor suppressor candidate 3; ER, endoplasmic reticulum.
