# Supplementary figures and images for: The LncRNA MIR503HG/miR-224-5p/TUSC3 Signaling Cascade Suppresses Gastric Cancer Development via Modulating ATF6 Branch of Unfolded Protein Response
Source: Front Oncol. 2021 Jul 26;11:708501. doi: 10.3389/fonc.2021.708501 (PMC8352579; doi:10.3389/fonc.2021.708501)

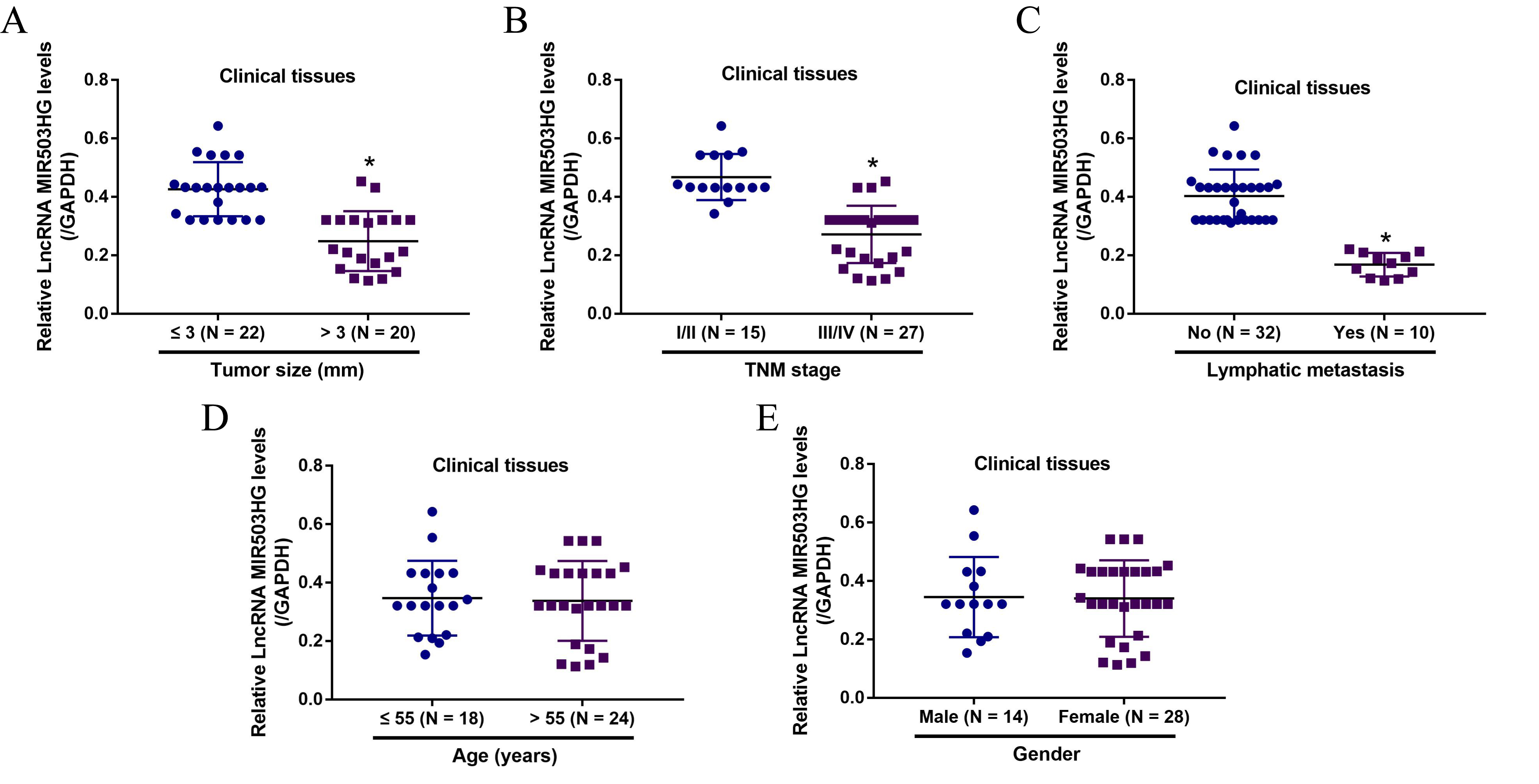

Supplement: Supplementary Figure 1 — Analysis of the correlations of LncRNA MIR503HG and the clinical characteristics in GC patients by Real-Time qPCR. Each experiment contained 3 repetitions, and P < 0.05 were marked by "*". [file Image_1.jpeg]

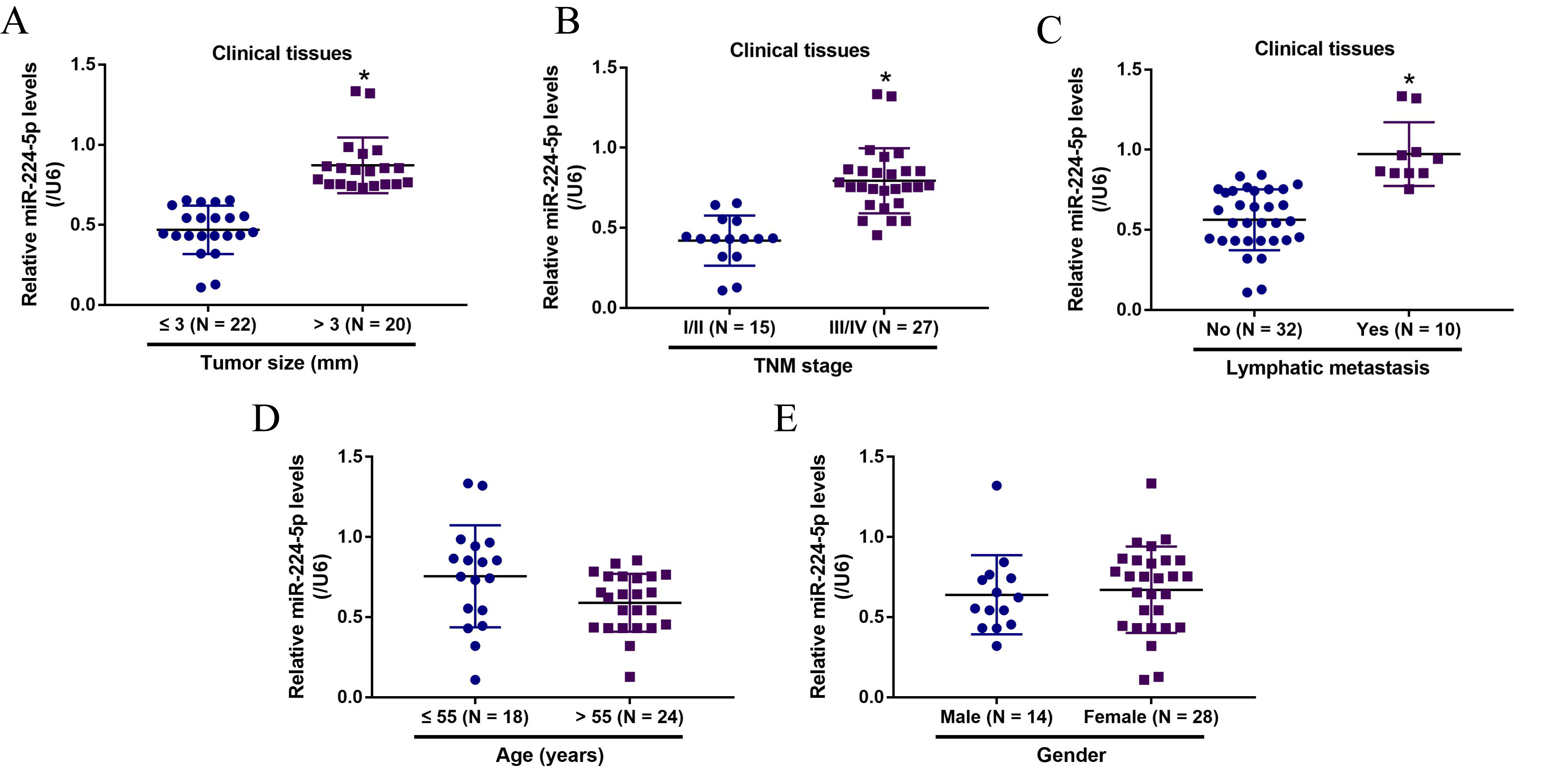

Supplement: Supplementary Figure 2 — Analysis of the correlations of miR-224-5p and the clinical characteristics in GC patients by Real-Time qPCR. Each experiment contained 3 repetitions, and P < 0.05 were marked by "*". [file Image_2.jpeg]

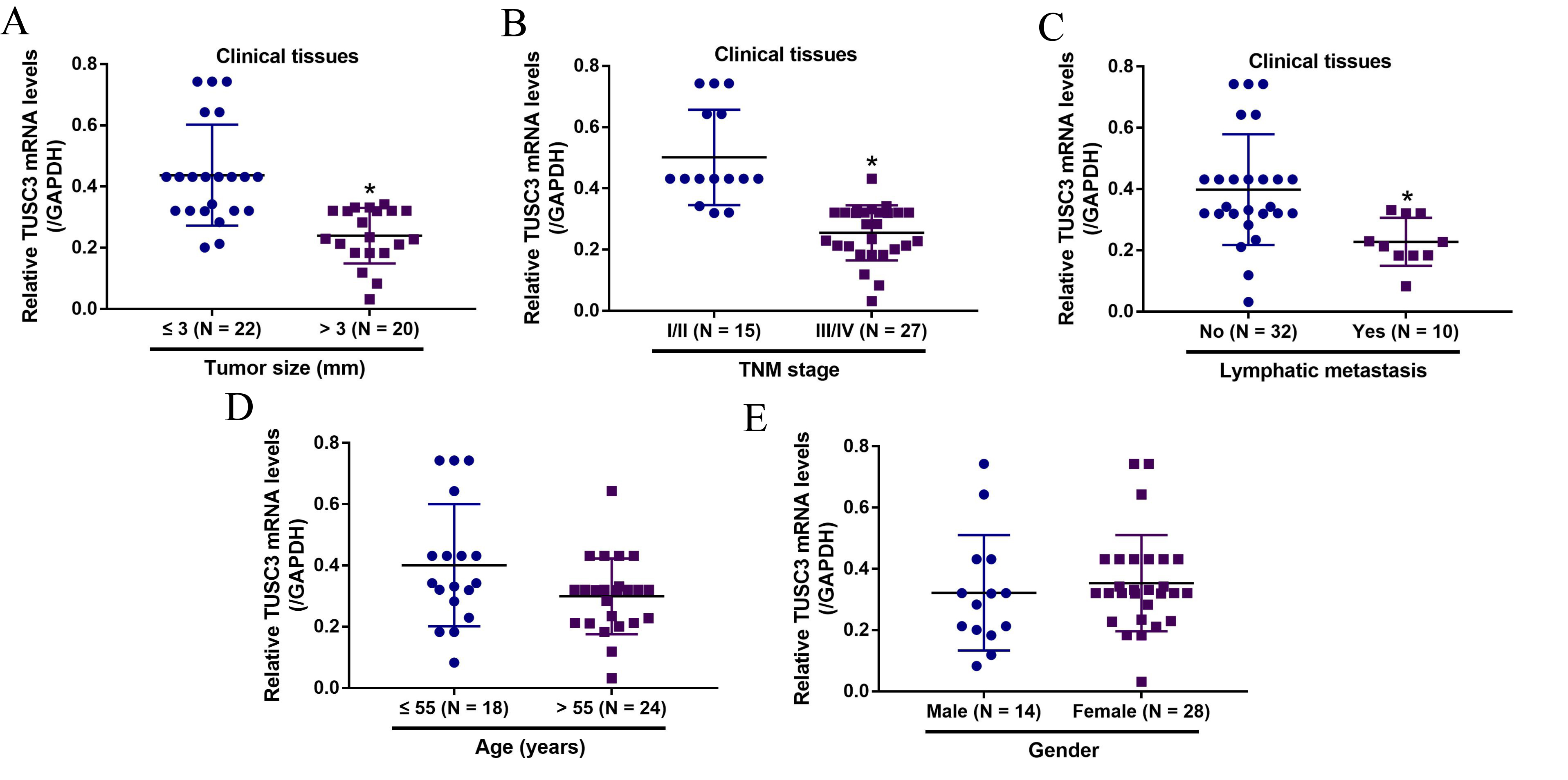

Supplement: Supplementary Figure 3 — Analysis of the correlations of TUSC3 mRNA and the clinical characteristics in GC patients by Real-Time qPCR. Each experiment contained 3 repetitions, and P < 0.05 were marked by "*". [file Image_3.jpeg]

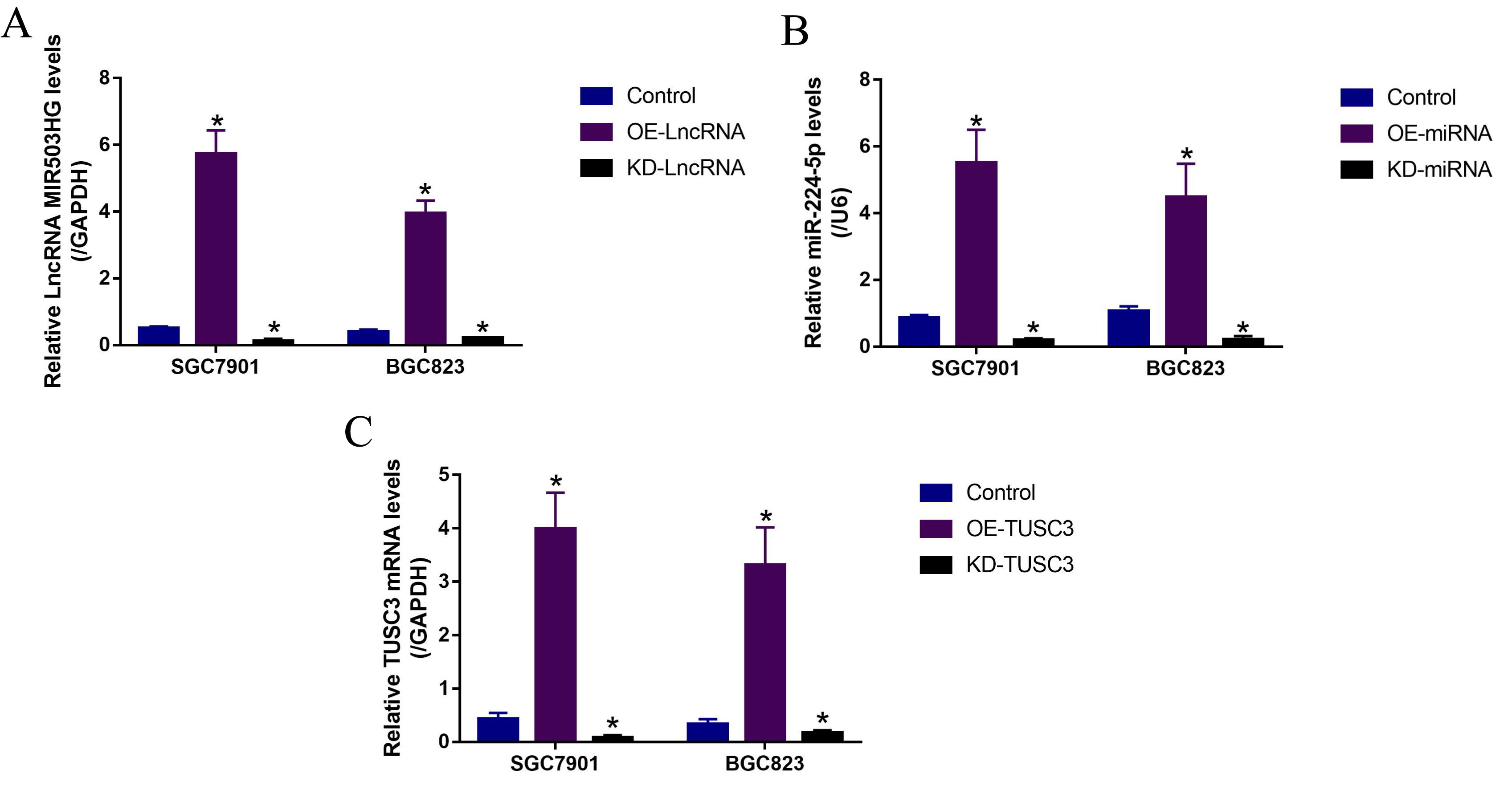

Supplement: Supplementary Figure 4 — LncRNA MIR503HG, miR-224-5p and TUSC3 were downregulated and overexpressed in GC cells, examined by Real-Time qPCR analysis. Each experiment contained 3 repetitions, and P < 0.05 were marked by "*". [file Image_4.jpeg]

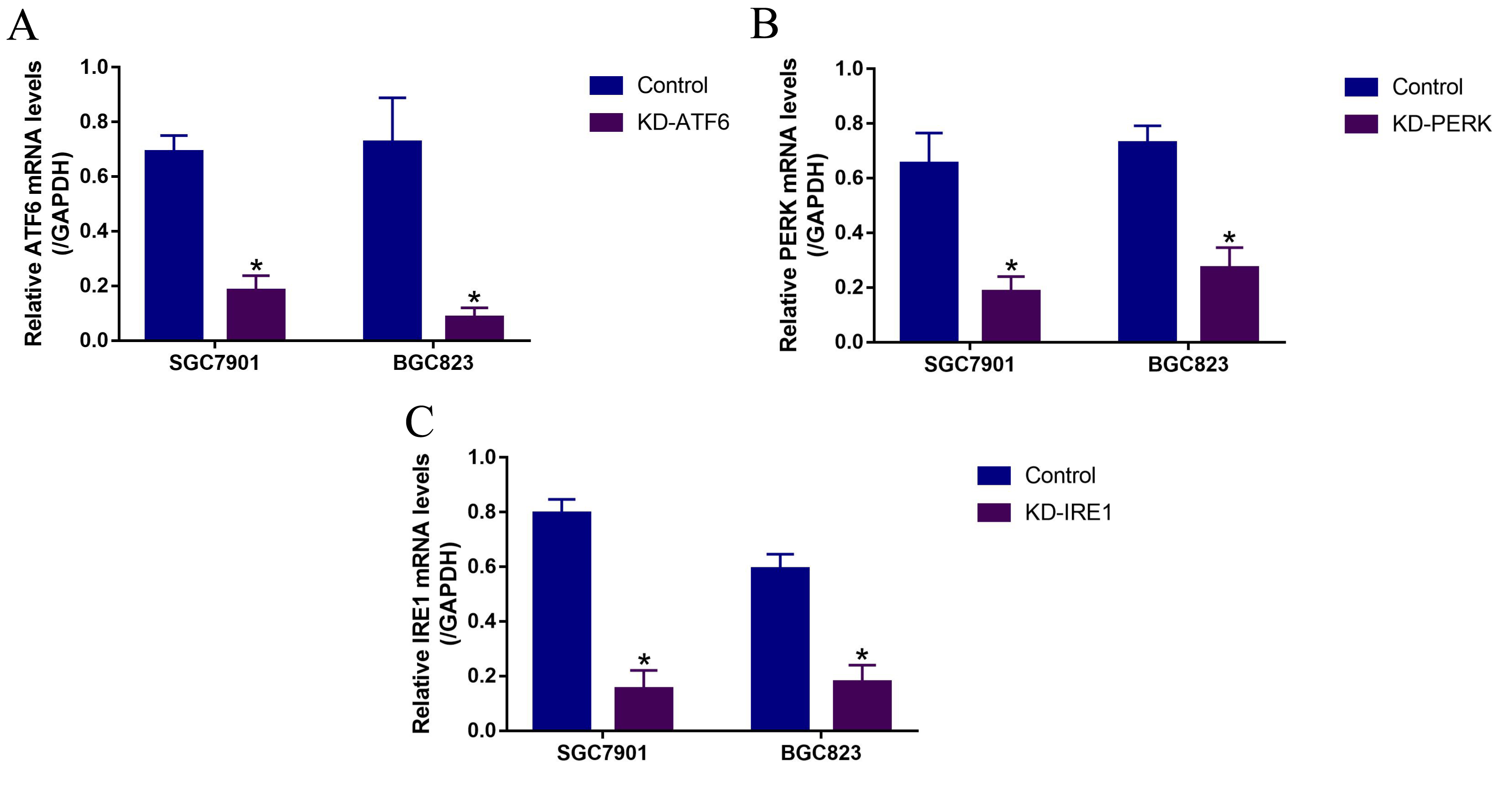

Supplement: Supplementary Figure 5 — Real-Time qPCR was used to determine that ATF6, PERK and IRE1 were successfully deleted in the GC cells. Each experiment contained 3 repetitions, and P < 0.05 were marked by "*". [file Image_5.jpeg]

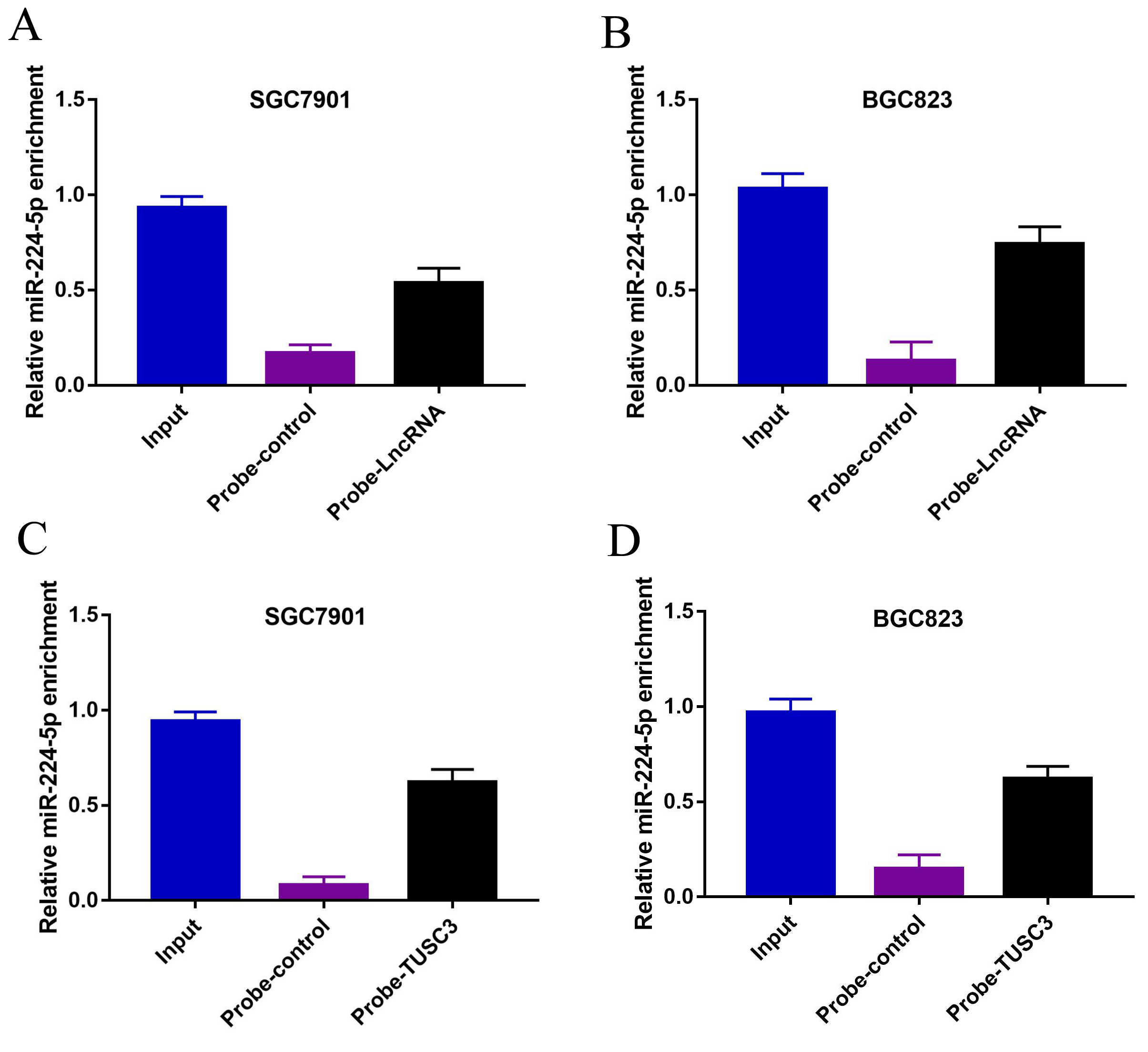

Supplement: Supplementary Figure 6 — RNA pull-down assay was performed to verify the targeting sites among LncRNA MIR503HG, miR-224-5p and 3’UTR of TUSC3 in GC cells. Each experiment contained 3 repetitions, and P < 0.05 were marked by "*". [file Image_6.jpeg]

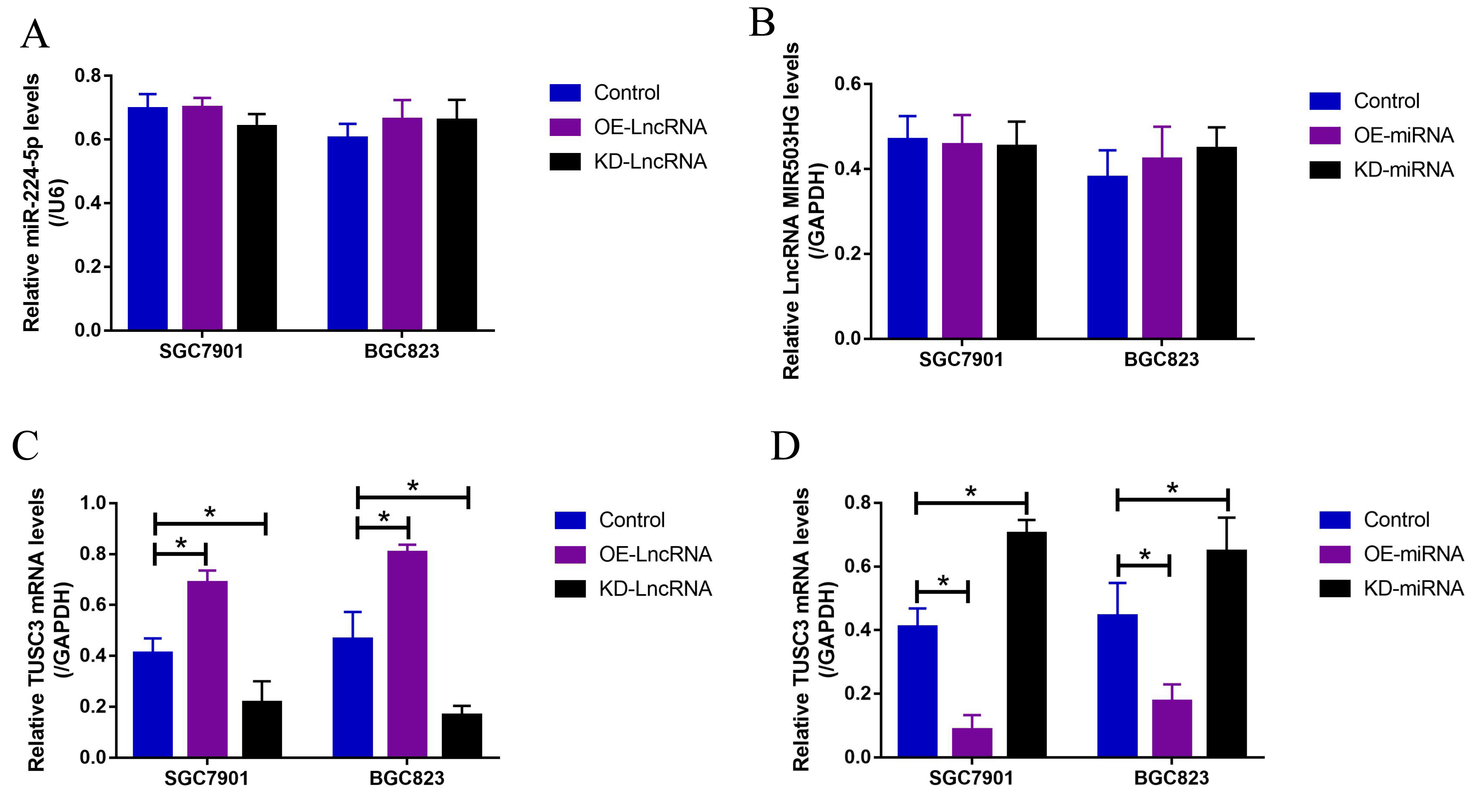

Supplement: Supplementary Figure 7 — The regulating effects among LncRNA MIR503HG, miR-224-5p and TUSC3 were measured by Real-Time qPCR analysis. The regulating effects of LncRNA MIR503HG on (A) miR-224-5p and (C) TUSC3 mRNA expressions. MiR-224-5p was overexpressed and downregulated, and its effects on (B) LncRNA MIR503HG and (D) TUSC3 mRNA were evaluated. Each experiment contained 3 repetitions, and P < 0.05 were marked by "*". [file Image_7.jpeg]
